# Large-Scale Social and Behavior Change Communication Interventions Have Sustained Impacts on Infant and Young Child Feeding Knowledge and Practices: Results of a 2-Year Follow-Up Study in Bangladesh

**DOI:** 10.1093/jn/nxy147

**Published:** 2018-08-29

**Authors:** Sunny S Kim, Phuong Hong Nguyen, Lan Mai Tran, Tina Sanghvi, Zeba Mahmud, Mohammad Raisul Haque, Kaosar Afsana, Edward A Frongillo, Marie T Ruel, Purnima Menon

**Affiliations:** 1 Poverty, Health, and Nutrition Division, International Food Policy Research Institute (IFPRI), Washington, DC; 2 Alive & Thrive, FHI 360, Hanoi, Vietnam; 3 Alive & Thrive, FHI 360, Washington, DC; 4 Alive & Thrive, FHI 360, Dhaka, Bangladesh; 5 Health, Nutrition and Population Programme, BRAC, Dhaka, Bangladesh; 6 Arnold School of Public Health, University of South Carolina, Columbia, SC; 7 Poverty, Health, and Nutrition Division, IFPRI, New Delhi, India

**Keywords:** Bangladesh, breastfeeding, complementary feeding, infant and young child feeding, sustainability

## Abstract

**Background:**

Sustained improvements in infant and young child feeding (IYCF) require continued implementation of effective interventions. From 2010–2014, Alive & Thrive (A&T) provided intensive interpersonal counseling (IPC), community mobilization (CM), and mass media (MM) in Bangladesh, demonstrating impact on IYCF practices. Since 2014, implementation has been continued and scaled up by national partners with support from other donors and with modifications such as added focus on maternal nutrition and reduced program intensity.

**Objective:**

We assessed changes in intervention exposure and IYCF knowledge and practices in the intensive (IPC + CM + MM) compared with nonintensive areas (standard nutrition counseling + less intensive CM and MM) 2 y after termination of initial external donor support.

**Methods:**

We used a cluster-randomized design with repeated cross-sectional surveys at baseline (2010, *n* = 2188), endline (2014, *n* = 2001), and follow-up (2016, *n* = 2400) in the same communities, among households with children 0–23.9 mo of age. Within-group differences over time and differences between groups in changes were tested.

**Results:**

In intensive areas, exposure to IPC decreased slightly between endline and follow-up (88.9% to 77.2%); exposure to CM activities decreased significantly (29.3% to 3.6%); and MM exposure was mostly unchanged (28.1–69.1% across 7 TV spots). Exposure to interventions did not expand in nonintensive areas. Most IYCF indicators in intensive areas declined from endline to follow-up, but remained higher than at baseline. Large differential improvements of 12–17 percentage points in intensive, compared with nonintensive areas, between baseline and follow-up remained for early initiation of and exclusive breastfeeding, timely introduction of foods, and consumption of iron-rich foods. Differential impact in breastfeeding knowledge remained between baseline and follow-up; complementary feeding knowledge increased similarly in both groups.

**Conclusions:**

Continued IPC exposure and sustained impacts on IYCF knowledge and practices in intensive areas indicated lasting benefits from A&T's interventions as they underwent major scale-up with reduced intensity. This trial was registered at clinicaltrials.gov as NCT02740842.

## Introduction

Appropriate infant and young child feeding (IYCF) practices are important for optimal child health, growth, and development (1–5). The evidence base on impacts of different combinations of interventions to achieve these recommended practices is growing (1, 2, 6–10). These interventions, although often successful, have been noted as generally failing to achieve and maintain scale (11–14). Also, sustained improvements in IYCF likely require continued implementation of effective interventions and sustained enabling social environments.

Recent impact evaluations of large-scale behavior-change communication (BCC) interventions to improve IYCF practices in several countries have shown that intensive interpersonal counseling (IPC) combined with mass media (MM) and community mobilization (CM) activities have positive impacts on breastfeeding (BF) (15, 16) and complementary feeding (CF) practices (15, 17, 18). In Bangladesh, combined intensive interventions resulted in significant improvements in exclusive breastfeeding (EBF) (by 36.2 percentage points—pp), early initiation of breastfeeding (EIBF) (by 16.7 pp) (16), and CF practices (16.3 pp, 14.7 pp, 22 pp, and 24.6 pp for minimum dietary diversity, minimum meal frequency, minimum acceptable diet, and consumption of iron-rich foods, respectively) (16, 17). Similar results were obtained in Vietnam (18); and patterns of increased EBF, EIBF, and multiple CF practices in intervention areas were observed in Ethiopia (15). Evaluations of other large-scale BCC interventions similarly showed a significant increase in EBF through BF counseling within a routine health service delivered by community health agents in Brazil (19) and improvements in both BF and CF practices through peer counseling by mother support groups in India (20).

With the growing evidence that BCC interventions can achieve scale and be effective, there is also a need to determine whether these achievements can be sustained, especially once initial external donor funding ends. What happens after seed money or initial project support and funding stop and whether program activities and effects are sustainable are of increasing interest among researchers, evaluators, funders, and community partners. Methodologically rigorous empirical studies of program sustainability are scarce (21, 22), however, and fraught with problems such as lack of unified definitions, approaches, and methods (23–28).

This paper assesses the sustainability of impacts among the target population group originally included in the evaluation of a large-scale BCC intervention program to improve IYCF practices, after termination of external funding from an initial donor agency. During the 2-y period between the end of the original program in 2014 and the follow-up study in 2016, significant programmatic changes occurred as implementation was taken up by national partners with support from new donors. First, the program was scaled up to >90% of the *upazilas* (subdistricts) in the country (from 10% during the original program); second, the intervention package introduced a new focus on maternal nutrition; and third, the program intensity was reduced. We refer to sustainability as “the extent to which an evidence-based intervention can deliver its intended benefits over an extended period of time, [particularly] after external support from the donor agency is terminated” (25, 29). In the context of our study, we describe the changes to the program that took place after the initial external funds expired and focus on the outcomes after that event. This paper reports on findings from a 2-y follow-up survey to a cluster-randomized impact evaluation of BCC interventions, to assess changes in intervention exposure and whether impacts on IYCF knowledge and practices were sustained.

## Methods

### 

#### Program description

In Bangladesh, from 2010–2014, the Alive & Thrive (A&T) initiative provided intensive IPC, CM, and MM at scale. Detailed descriptions of these interventions have been provided elsewhere (16, 17, 30). The community-based IPC during home visits and CM activities were delivered by BRAC, a large nongovernmental organization, in 50 rural subdistricts through its extensive cadres of frontline workers involved in its existing Essential Health Care program, intended to achieve and sustain large-scale delivery of interventions. Standard nutrition counseling was delivered during routine home visits by BRAC health workers (called *Shasthya Kormi*) and community volunteers (called *Shasthya Sebika*) in the nonintensive intervention areas. In the intensive intervention areas, a new cadre of nutrition-focused frontline workers, the *Pushti Kormi* (nutrition promoters), together with the community volunteers, conducted multiple age-targeted IYCF-focused visits to households with pregnant women and mothers of children ≤2 y of age. The community volunteers and nutrition promoters were supervised regularly, and they received performance-based cash incentives (US$6–8/mo) for tasks which included ensuring high coverage, carrying out age-appropriate counseling at home visits, and collecting maternal reports of practicing the recommended behaviors. In the intensive areas, CM included sensitization of community leaders to IYCF, and community video shows and theater shows focused on IYCF. The MM component consisted of 7 nationally broadcast TV spots with messages on various aspects of IYCF; 2 spots focused on BF, 4 spots were related to CF, and 1 spot was on handwashing. Media dark strategies such as screening of TV spots within communities where TV reach was lower were implemented. In nonintensive areas, there was standard CM through local meetings on various health topics and there were no media dark strategies for MM. Over the 4-y period, the combined interventions led to large significant impacts on IYCF practices (16, 17).

In 2014, external funding support from the initial donor agency ended, but BRAC continued to deliver the IPC and CM activities with several modifications (**Table 1**). With the support of new external donor funds, BRAC expanded delivery of IPC on IYCF to 456 out of 490 total subdistricts in the country, including the intensive and nonintensive areas. There was an added focus on maternal nutrition during IPC, less frequent training and supervision of frontline workers, and there were reduced performance-based incentives for volunteer workers (31). Although BRAC health workers and community volunteers continued to deliver IYCF counseling and support services, nutrition promoters were discontinued and their functions were taken up by BRAC health workers who were designated to deliver nutrition interventions after 2014. CM activities focused on IYCF were reduced from 5 sessions to 1 session per year. The MM campaign lapsed after 2014, but the Government of Bangladesh adopted the A&T MM materials and started broadcasting the IYCF videos again nationally in early 2016.

**TABLE 1:** Summary of intensive interventions in 2010–2014 and modifications after 2014^1^

Intervention	2010–2014	After 2014
Interpersonal counseling		
Coverage area	50 A&T *upazilas* (funded by BMGF)	456 *upazilas* in 61 districts (funded by DFID and AusAID) and additional 26 *upazilas* (funded by USAID)
Technical content	IYCF information	IYCF information
		Information about dietary diversity and additional food for pregnant and lactating women
Frequency of contact	SS: monthly visits	SS: monthly visits
	SK: 12 visits	SK-Pushti: 12 visits + 3 additional visits during pregnancy
	PK: 8 visits	PKs changed to SK-Pushti
Training	Monthly refreshers by BRAC staff	Quarterly refreshers in 2015
		Stopped refresher training in 2016 (SKs meet monthly to review)
Monitoring and supervision	1 monitor for 2 *upazilas*	1 monitor for 20 *upazilas*
Performance-based incentives for volunteer workers	Incentive criteria: EIBF, EBF, timely introduction to complementary foods, meal frequency, amount, density, ASF, handwashing, and sick child feeding for <2-y-olds	Incentive criteria reduced to 5 indicators: EIBF, EBF, timely introduction to complementary foods, handwashing, and identification of pregnancy
	20 Taka per indicator	10–15 Taka, depending on indicator
Community mobilization	5 sessions in each *upazila*/y	1 session in each *upazila*/y
	Video shows, popular theater shows, and doctors’ seminars	No video shows, popular theater shows, or doctors’ seminars
		3 sessions in each *upazila*/y about homesteading garden (multisectoral approach)
Mass media campaign	TV spots 1–7	Government broadcasts TV spots starting in early 2016
	Media dark strategies	Media dark strategies discontinued

^1^ASF, animal source foods; AusAID, Australian Agency for International Development; A&T, Alive & Thrive; BMGF, Bill & Melinda Gates Foundation; DFID, Department for International Development of the United Kingdom; EBF, exclusive breastfeeding; EIBF, early initiation of breastfeeding; IYCF, infant and young child feeding; PK, Pushti Kormi (nutrition promoter); SK, Shasthya Kormi (health worker); SS, Shasthya Sebika (health volunteer); USAID, United States Agency for International Development.

The modifications made to the intensive program after 2014, when external funds from the original donor phased out and new funding from other donors became available, have implications for the definition of sustainability and our evaluation. The changes post-2014 involved 2 major components: *1)* large scale-up of the program; and *2)* implementation of programmatic changes, which included both a reduction in intensity and an expansion of the intervention focus. All original study areas were included in these changes, but they were affected in different ways and degrees as shown in our study results.

#### Study design

This study applied the same design as the cluster-randomized nonblinded impact evaluation to compare the impact of the 2 A&T intervention packages: intensive consisting of intensified IPC, CM, and MM; and nonintensive consisting of standard IPC and less intensive CM and MM. Among the 50 rural subdistricts where BRAC implemented the program, 20 subdistricts (clusters) were randomly selected for inclusion in the evaluation sample before baseline and then randomized to 2 intervention groups (10 intensive and 10 nonintensive). Cross-sectional household surveys were conducted at baseline (2010), exactly 4 y later at endline (2014), and another 2 y later (same time of year) at follow-up (2016) in the same communities among households with children 0–23.9 mo of age, which are the target population for the nutrition interventions. We considered the time period between endline and follow-up as the program sustainability period for the purposes of this study, although sustainability continues beyond the duration of this study.

#### Sample size estimations

The same sample size estimations were used as for the 2014 endline survey to determine whether the impacts achieved since baseline were sustained at follow-up in 2016. There were 2 study samples: *1)* households with children 0–5.9 mo of age for BF-related indicators, and *2)* households with children 6–23.9 mo of age for CF-related indicators. Sample sizes were estimated to detect differences in the primary outcomes (i.e., EBF and minimum dietary diversity) between the 2 intervention groups, considering alpha of 0.05, power of 0.80, intraclass correlation of 0.01, and baseline prevalence of the primary outcomes of 43% for EBF and 44% for minimum dietary diversity. We estimated that sample sizes of 980 children aged 0–5.9 mo (490 per group) and 980 children 6–23.9 mo (490 per group) were sufficient to detect a ≥10 pp difference in the proportion of children achieving EBF and minimum dietary diversity, respectively, between baseline and follow-up.

#### Outcomes

The primary outcomes were IYCF practices in children 0–23.9 mo of age based on the WHO recommended indicators (32). Eight core IYCF indicators were examined: *1)* EIBF (defined as the percentage of children aged 0–23.9 mo who were put to the breast within 1 h of birth); *2)* EBF (defined as the percentage of children 0–5.9 mo who were fed only breast milk in the previous 24 h); *3)* continued BF at 1 y (defined as the percentage of children 12–15.9 mo who are fed breast milk); *4)* introduction of solid, semi-solid, or soft foods (SSSF, among children aged 6–8.9 mo); *5)* minimum dietary diversity (defined as the consumption of foods from ≥4 of 7 food groups in the previous 24 h by children aged 6–23.9 mo); *6)* minimum meal frequency (defined as the frequency of consuming foods as appropriate for age and BF status by children aged 6–23.9 mo); *7)* minimum acceptable diet (defined as the achievement of the minimum dietary diversity and age-appropriate minimum meal frequency, as defined previously, by children aged 6–23.9 mo); and *8)* consumption of iron-rich food (by children aged 6–23.9 mo) (33). The IYCF indicators were constructed based on maternal previous-day recall of liquids and foods consumed by the target child.

The secondary outcomes were maternal knowledge about BF and CF, assessed based on mothers’ responses to a set of 8 questions related to BF (e.g., how soon after birth BF should be initiated, whether colostrum should be given to the baby, benefits of EBF, the ideal duration of BF, whether mothers should continue BF if they become sick or pregnant) and 8 questions about CF (e.g., when liquids other than breast milk and foods should be introduced, frequency of giving meals at different ages, how to feed a sick child during and after illness). Some items were validated in a previous study (34) and adapted for our study context, and others were added to reflect information provided in the interventions. The same questions were included in all 3 survey rounds. Each knowledge item was given a score of 1 (correct) or 0 (incorrect), and the sum was used as the BF and CF knowledge scores (scale: 0–8). Intervention exposure was measured by maternal recall of home visits (by BRAC frontline workers) received in the last 6 mo, number of times visited, attendance at a CM activity in the last 1 y, and recall of ever having seen the A&T TV spots.

#### Statistical analysis

Differences in the sample characteristics between the 2 intervention groups by survey round were tested with the use of linear (for continuous variables) or logit (for categorical variables) regression models accounting for geographical clustering. For the analyses of outcome indicators, we estimated the differences over time (from endline to follow-up) by intervention group via regression models while adjusting for geographical clustering, infant age, and sex. This allowed us to describe and compare patterns of change in intervention exposure, knowledge, and practices during the sustainability period in the 2 intervention areas. Then, to determine whether impacts achieved at endline (from baseline to endline) were sustained at follow-up (from baseline to endline), we derived difference-in-difference impact estimates (DDEs), through the use of fixed-effects regression models that assessed differences between the 2 intervention groups over time. We presented the intention-to-treat DDEs, adjusted for geographical clustering, infant age and sex, and factors that differed at baseline, endline, or follow-up. Data analysis was performed through the use of Stata 13 (StataCorp).

#### Ethical approval

The research protocol received ethical clearance from the Bangladesh Medical Research Council and the Institutional Review Board at the International Food Policy Research Institute. The evaluation is registered with the Clinical Trials Registry at clinicaltrials.gov (NCT02740842). All mothers of study children were provided with detailed information about the study at recruitment. Verbal informed consent was obtained from mothers before their participation in the survey.

## Results

### 

#### Trial flow and intervention timeline

**FIGURE 1:**
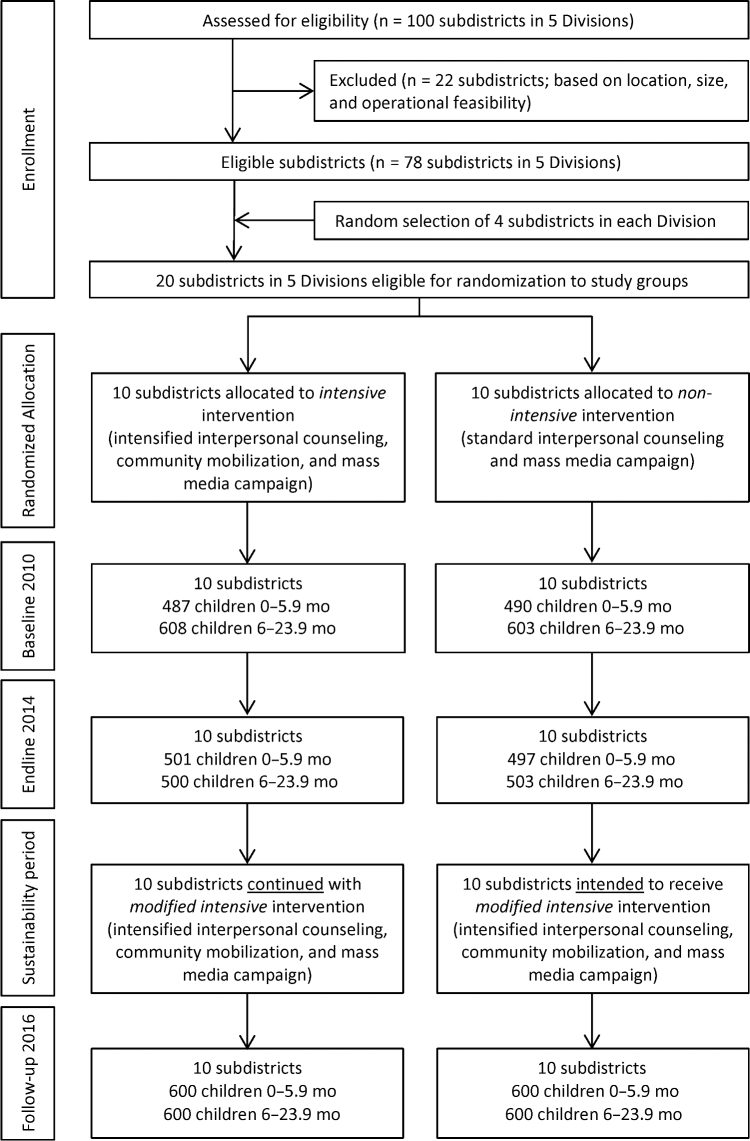
Trial profile.

No study clusters were lost to follow-up across the different survey rounds (**Figure 1**), and there was little variation in cluster size across intervention group and over time.

#### Sample characteristics

There were no significant differences in most child, maternal, and household characteristics between intervention groups across the survey rounds (**Table 2**). At follow-up, mothers living in the intensive area were slightly older (by less than one-half of a year) compared with those in the nonintensive areas. More households in the intensive area owned a home garden or agricultural land, and were more food secure, compared with those in nonintensive areas.

**TABLE 2:** Selected characteristics of the study sample for children aged 0–23.9 mo, by program group and survey round^1^

	Baseline 2010		Endline 2014		Follow-up 2016	
	Intensive	Nonintensive	Intensive	Nonintensive	Intensive	Nonintensive
Characteristics	(*n* = 1095)	(*n* = 1093)	(*n* = 1001)	(*n* = 1000)	(*n* = 1200)	(*n* = 1200)
Household factors						
Children aged <5 y, *n*	1.3 ± 0.5	1.3 ± 0.5	1.3 ± 0.5	1.3 ± 0.5	1.4 ± 0.6	1.4 ± 0.6
Female household head, %	14.3	9.5	12.3	11.1	4.3	3.0
Ownership of house, %	94.1	94.7	96.2	94.5	99.6	99.1
Ownership of garden, %	29.22	31.66	31.0	35.5	27.9***	14.8
Ownership of agricultural land, %	49.5*	40.9	51.6	45.8	51.9*	45.3
Food insecurity,^2^ %	31.7	30.6	14.2*	20.8	25.2**	36.1
Dietary diversity (range: 0–12), *n*	7.6 ± 1.8	7.7 ± 1.7	8.1 ± 1.8	7.9 ± 1.7	8.8 ± 1.4	8.7 ± 1.4
Maternal factors						
Age, y	26.0 ± 6.1	25.6 ± 5.8	25.3 ± 5.4*	24.7 ± 5.3	25.3 ± 5.3*	24.9 ± 5.3
Schooling, y	4.8 ± 3.5	5.1 ± 3.6	5.8 ± 3.3	6.0 ± 3.3	6.3 ± 3.1	6.4 ± 3.2
Occupation as housewife, %	96.3	93.8	76.2	84.1	92.1	94.4
Child factors						
Age, mo	9.8 ± 7.0	9.7 ± 7.0	9.0 ± 6.7	8.9 ± 6.8	8.8 ± 6.8	8.6 ± 6.6
Female, %	49.2	50.1	51.8	50.1	48.8	51.5

^1^Values are means ± SDs or percentages. *,**,***Different from nonintensive at that time: *P < 0.05, **P < 0.01, ***P < 0.001.

^2^Household food insecurity was measured by the use of the Food and Nutrition Technical Assistance III/US Agency for International Development's Household Food Insecurity Access Scale.

#### Intervention exposure

In intensive areas, exposure to BRAC frontline workers for IPC in the last 6 mo and the frequency of home visits decreased significantly between endline and follow-up, but the exposure remained moderately high (59.3% for home visits by community volunteers and 77.2% for home visits by nutrition promoters) (**Table 3**). In the nonintensive areas, there was no change in exposure or frequency of home visits at follow-up; there was no indication of expansion of intensive IPC into the nonintensive areas. In relation to counseling quality, there was a decrease in the duration but an increase in discussion about IYCF in the last visits made by frontline workers in the intensive areas (**Supplemental Table 1**). In the nonintensive areas, more mothers reported discussing IYCF during the last visit by health workers at follow-up.

**TABLE 3:** Exposure to interpersonal counseling by frontline workers among mothers with children aged 0–23.9 mo, by program group and survey round^1^

	Endline 2014 (T2)		Follow-up 2016 (T3)			
	Intensive	Nonintensive	Intensive	Nonintensive	Intensive	Nonintensive
Indicator	(*n* = 1001)	(*n* = 1000)	(*n* = 1200)	(*n* = 1200)	T3 – T2	T3 – T2
Visited by SS in the last 6 mo, %	87.4***	17.4	59.3***	12.3	−28.1^###^	−5.1
Visited by SK in the last 6 mo, %	29.9	24.0	39.9**	16.6	10.0	−7.4^#^
Visited by PK in the last 6 mo, %	88.9***	0.0	77.2***	0.1	−11.7^#^	0.1
Visits by SS in the last 6 mo, *n*	4.3 ± 2.9***	0.6 ± 1.5	2.0 ± 2.3***	0.3 ± 1.2	−2.3^##^	−0.3
Visits by SK in the last 6 mo, *n*	1.0 ± 2.0*	0.6 ± 1.3	1.2 ± 1.8**	0.4 ± 1.0	0.2	−0.2
Visits by PK in the last 6 mo, *n*	3.5 ± 2.1***	0.0	2.6 ± 2.2***	0.0	−0.9^##^	0.0

^1^Values are means ± SDs or percentages. *,**,***Different from nonintensive at that time: *P < 0.05, **P < 0.01, ***P < 0.001. ^#,##,###^Significant change from endline to follow-up: ^#^*P* < 0.05, ^##^*P* < 0.01, ^###^*P* < 0.001. PK, Pushti Kormi (nutrition promoter); SK, Shasthya Kormi (health worker); SS, Shasthya Sebika (health volunteer); T, time.

Between endline and follow-up in intensive areas, exposure to CM activities decreased significantly (29.3% to 3.6% for video shows), whereas MM exposure remained unchanged and moderate (28.1–69.1% across 7 TV spots), except for a significant decrease in exposure to TV spot 3 (on the importance of good nutrition for brain development) (**Table 4**). There was no significant change in exposure to CM or MM between endline and follow-up in the nonintensive areas.

**TABLE 4:** Exposure to community mobilization activities and mass media among mothers with children aged 0–23.9 mo, by program group and survey round^1^

	Endline 2014 (T2)		Follow-up 2016 (T3)			
	Intensive	Nonintensive	Intensive	Nonintensive	Intensive	Nonintensive
Indicator	(*n* = 1001)	(*n* = 1000)	(*n* = 1200)	(*n* = 1200)	T3 – T2	T3 – T2
Exposure to CM						
Watched video show in last 1 y	29.3***	0.8	3.6**	0.8	−25.7^#^	0.0
Attended popular theatre in last 1 y	13.8***	0.5	7.2**	0.6	−6.6^#^	0.1
Exposure to MM^2^						
Ever watched TVC1	67.9	61.3	67.1	59.8	−0.8	−1.5
Ever watched TVC2	64.6	56.3	62.3	53.8	−2.3	−2.5
Ever watched TVC3	47.9*	33.6	28.1	22.6	−19.8^#^	−11.0
Ever watched TVC4	66.7	60.6	69.1	60.3	2.4	−0.3
Ever watched TVC5	58.8	48.6	51.7	44.7	−7.1	−3.9
Ever watched TVC6	58.2	48.2	58.3	49.8	0.1	1.6
Ever watched TVC7	61.8*	51.2	59.8	53.0	−2.0	1.8

^1^Values are percentages. *,**,***Different from nonintensive at that time: *P < 0.05, **P < 0.01, ***P < 0.001. ^#^Significant change from endline to follow-up: ^#^*P* < 0.05. CM, community mobilization; MM, mass media; T, time; TVC, television commercial/spot.

^2^TVC1, early initiation of breastfeeding; TVC2, exclusive breastfeeding; TVC3, importance of good nutrition for brain development; TVC4, feeding animal source food; TVC5, frequency and quantity of complementary feeding; TVC6, feeding children with poor appetite; TVC7, handwashing.

#### Change over time and sustained impacts on IYCF practices and knowledge

**FIGURE 2:**
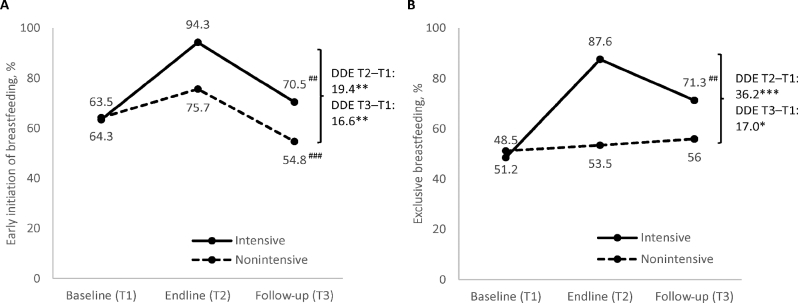
Breastfeeding practices, by program group and survey round. (A) Early initiation of breastfeeding and (B) exclusive breastfeeding. *,**,***Significantly different DDEs with clustered SEs comparing intensive and nonintensive areas from baseline to endline or baseline to follow-up, accounting for geographic clustering at *upazila* and district levels. *P < 0.05, **P < 0.01, ***P < 0.001. ^##,###^Significant change from endline to follow-up: ^##^*P* < 0.01, ^###^*P* < 0.001. DDE, difference-in-difference estimate; T, time.

Apart from continued BF at 1 y, which remained high (>90%) over time in both intensive and nonintensive areas (**Supplemental Table 2**), prevalence of BF indicators decreased between endline and follow-up but remained higher than at baseline in the intensive areas (**Figure 2**). EIBF decreased by >20 pp in both groups; EBF decreased by 16.3 pp in intensive areas only, with no significant change in nonintensive areas. However, significant differential impacts between groups were sustained, i.e., DDE: 16.6 pp for EIBF and 17 pp for EBF between baseline and follow-up, respectively.

**FIGURE 3:**
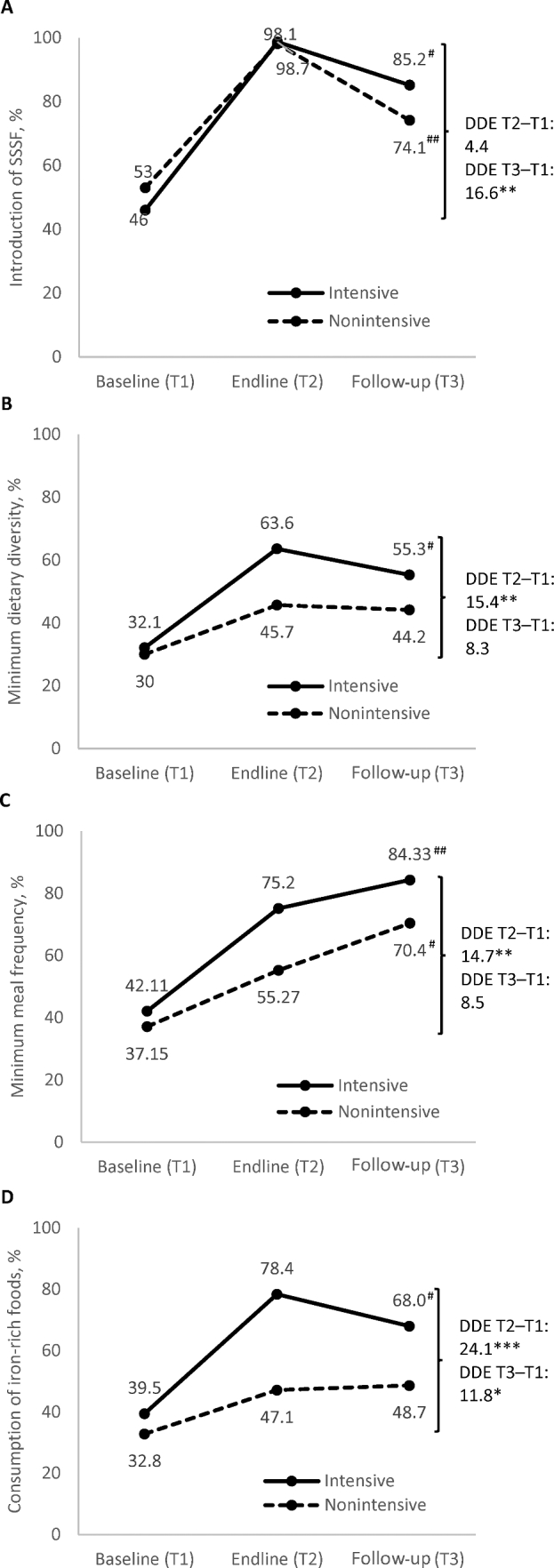
Complementary feeding practices, by program group and survey round. (A) Introduction of SSSF, (B) minimum dietary diversity, (C) minimum meal frequency, and (D) consumption of iron-rich foods. *,**,***Significantly different DDEs with clustered SEs comparing intensive and nonintensive areas from baseline to endline or baseline to follow-up, accounting for geographic clustering at *upazila* and district levels. *P < 0.05, **P < 0.01, ***P < 0.001. ^#,##^Significant change from endline to follow-up: ^#^*P* < 0.05, ^##^*P* < 0.05. DDE, difference-in-difference estimate; SSSF, solid, semi-solid, or soft food; T, time.

Patterns varied for CF practices (**Figure 3**). In intensive areas, timely introduction of SSSF, minimum dietary diversity, and consumption of iron-rich foods decreased significantly between endline and follow-up but remained higher than at baseline. In nonintensive areas, timely introduction of SSSF also decreased, whereas minimum dietary diversity and consumption of iron-rich foods remained unchanged. In contrast, minimum meal frequency increased significantly in both intensive and nonintensive areas. Sustained impacts were observed between baseline and follow-up for timely introduction of SSSF (although no significant impact was observed between baseline and endline) and consumption of iron-rich foods (DDE: 16.6 pp and 11.8 pp, respectively).

**FIGURE 4:**
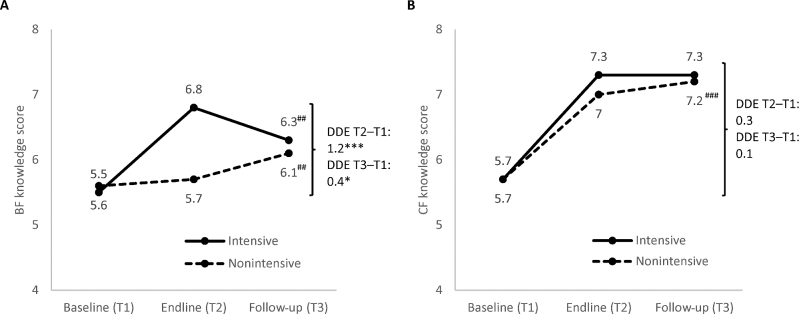
BF (A) and CF (B) knowledge scores among mothers with children aged 0–23.9 mo, by program group and survey round. *,***Significantly different DDEs with clustered SEs comparing intensive and nonintensive areas from baseline to endline or baseline to follow-up, accounting for geographic clustering at *upazila* and district levels. *P < 0.05, ***P < 0.001. ^##,###^Significant change from endline to follow-up: ^##^*P* < 0.01, ^###^*P* < 0.001. BF, breastfeeding; CF, complementary feeding; DDE, difference-in-difference estimate; T, time.

In relation to maternal IYCF knowledge, overall BF knowledge score decreased between endline and follow-up in intensive areas, but increased in nonintensive areas. The overall CF knowledge score remained the same between endline and follow-up in intensive areas and increased in nonintensive areas (**Figure 4**, **Supplemental Tables 3 and 4**). There was a small but significant sustained impact in BF knowledge between baseline and follow-up (DDE: 0.4 pp), but no significant sustained impact in CF knowledge.

## Discussion

At 2 y after endline, intervention exposure had decreased, but home visits by frontline workers for IPC remained moderately high in the intensive areas and significantly higher compared with the nonintensive areas. This finding suggests that BRAC has been able to maintain the contacts between its frontline workers and the target population in the intensive areas through its permanent Essential Health Care program platform. Modifications such as the shift in intervention focus, less intensive support to frontline workers (less frequent refresher training and monitoring), and reduced incentives for volunteers likely contributed to the decreased exposure. Exposure to CM activities decreased significantly, whereas MM exposure was mostly unchanged. There was also no indication of expansion of the intensive interventions (intensified IPC and CM) in the nonintensive areas at the follow-up time. Our findings on decreased intervention exposure corroborate some deviations from the original program design as further described in a separate study that assessed organizational sustainability (extent of program adaptation by local implementing partners and other stakeholders) (31).

Most IYCF indicators decreased at follow-up in the intensive areas compared with endline, but were still higher than at baseline, even after controlling for all factors that were different between groups at baseline, endline, or follow-up. With EIBF, we observed a similar decline in both intensive and nonintensive areas after endline. During this period, we also observed a significant increase in institutional delivery by 6.9–13 pp in both areas and an increase in the report of cesarean deliveries, which had increased significantly by 6 pp (from 23.5% to 29.5%), particularly in intensive areas (results not shown). Cesarean delivery is a significant barrier to EIBF, by delaying the initiation of BF compared with women giving birth vaginally (35), thus it is plausible that EIBF was influenced by these changes in the mode of delivery alongside the reduced intensity of interventions. Still, there were differential impacts between intervention groups sustained through follow-up for EIBF, EBF, introduction to SSSF, and consumption of iron-rich foods. These results are consistent with the sustainability literature that shows diminution of activities and effects almost immediately after initial project termination or external support expires; partial sustainability or continuation of some parts of a program or interventions and benefits is often noted (21, 22). However, the sustained impacts in the primary outcomes between baseline and follow-up lend evidence that there are continued benefits in the intensive interventions, because the IYCF practices improved over time in the intensive areas and above the difference over time in nonintensive areas, where we had observed no change in intervention exposure.

In the nonintensive areas, levels of IYCF indicators either decreased or remained steady between endline and follow-up, except for minimum meal frequency which increased similarly as in the intensive areas. Most of these results are in line with intervention exposure in this group, which remained mostly unchanged over time as mentioned above. For minimum meal frequency, the increase in both groups may be due to large increased knowledge specifically about age-appropriate meal frequency, as shown in the corresponding items that make up the CF knowledge score (Supplemental Table 4). BF and CF knowledge also increased in the nonintensive areas, despite the lack of evidence of expanded interventions in these areas. Mothers in the nonintensive areas likely received IYCF information from sources other than BRAC frontline workers and the MM intervention. In a separate paper on information diffusion based on our study data (36), we reported that awareness of IYCF messages increased significantly over time in nonintensive areas. Although BRAC workers continued to be the main source of IYCF information in intensive areas, other sources such as family members significantly increased between endline and follow-up in both intensive and nonintensive areas; other non-BRAC health workers also increased as a major source of IYCF information in nonintensive areas. Thus, wider diffusion of IYCF information through mothers’ social networks in the nonintensive areas seemed to have contributed to increased knowledge and awareness. Although knowledge is an important determinant of practice, the gap between knowledge and practice related to child feeding persists, pointing to the need for continued efforts in reducing sociocultural barriers and creating enabling environments for optimal feeding practices.

Given the need to maintain appropriate IYCF practices among new cohorts of infants and young children, it is important to sustain effective BCC interventions until these practices are incorporated as the norm among the target population. Behavior change is a dynamic process that requires time from contemplating the behavior to achieving maintenance and needs to be reinforced over longer periods of time (37). Optimal IYCF involves a complex set of behaviors within specific and narrow age periods that may require greater time and reinforcement to be maintained. This is supported by our observation of the same patterns of decline in most IYCF knowledge and practices among the subsample of mothers in our study who were potentially previously exposed to the A&T interventions between baseline and endline (∼45% of our study sample, data not shown). This finding of no difference in IYCF knowledge and practices between previously exposed and non–previously exposed mothers suggests that there is a continued need for reinforcing the behavior-change messages and supporting practices in this context.

Our study had several limitations. First, measurement of IYCF practices was based on maternal recall, which may be susceptible to social desirability bias in reporting. As reported in the impact evaluation, however, we found no differential results (between intensive and nonintensive groups) in reporting of IYCF practices due to respondents’ desire for social approval (16, 17). Second, our study was carried out only 2 y after endline of the impact evaluation and termination of support from the initial donor agency, which may be considered as a relatively short time period. Nevertheless, this period was sufficient to examine how intervention exposure changed even shortly after endline as well as to assess IYCF-related outcomes among new cohorts of children <2 y old. Further research on the effects of longer duration of sustainability is needed. Third, multiple changes across various implementation domains (training, supervision, monitoring, technical focus, incentive structure, etc.) took place since 2014, and we cannot disentangle which of those changes was most responsible for the sustainability of exposure and impacts. These are important implementation research questions and could be studied in the future. Fourth, our study of sustained outcomes was focused on the effects among the target population of the nutrition interventions. We did not examine the policy and regulatory institutions or organizational levels in connection to sustained service delivery, which was undertaken by a separate study. Nevertheless, the evidence linking intervention exposure to knowledge and practices in our study permitted interpretation of why the differences in outcomes were observed.

In conclusion, our study contributes to filling the evidence gap in methodologically rigorous empirical studies of program sustainability. This study offers evidence of decreased yet sustained impacts on IYCF practices even under modifications, suggesting program drift [i.e., gradual deviation of a program from its original design, as it is implemented and altered in response to new conditions (38)]. The reduced fidelity to the intervention, as compared with the intended design and outcomes (39), contributed to lower benefits. Still, continued IPC exposure and sustained impacts on IYCF practices in intensive areas in 2016 indicate lasting benefits from A&T's interventions, as they underwent major scale-up and adaptations after termination of initial external donor support. Thus, large benefits were achieved even with reduced intervention intensity, warranting the need for continued support and effort to expand and sustain the proven effective interventions that were achieved during the A&T project period and to continue supporting appropriate IYCF practices.

## Supplementary Material

Supplemental File

## Notes

Supported by the Bill & Melinda Gates Foundation (OPP50838), through Alive & Thrive, managed by FHI 360; additional financial support to the evaluation study was provided by the CGIAR Research Program on Agriculture for Nutrition and Health (A4NH), led by the International Food Policy Research Institute.

Supplemental Tables 1–4 are available from the “Supplementary data” link in the online posting of the article and from the same link in the online table of contents at https://academic.oup.com/jn/.

Author disclosures: TS, ZM, MRH, and KA were members of the program implementation team that designed and implemented the interventions studied in this manuscript. They reviewed the manuscript and provided interpretation of the results, but final decisions for content lay with the primary authors from the evaluation team (SSK, PHN, EAF, MTR, and PM). SSK, PHN, LMT, TS, ZM, MRH, KA, EAF, MTR, and PM, no conflicts of interest.

**Abbreviations used**
A&T: Alive & Thrive
BCC: behavior-change communication
BF: breastfeeding
CF: complementary feeding
CM: community mobilization
DDE: difference-in-difference impact estimate
EBF: exclusive breastfeeding
EIBF: early initiation of breastfeeding
IPC: interpersonal counseling
IYCF: infant and young child feeding
MM: mass media
pp: percentage point
SSSF: solid, semi-solid, or soft food
