# Supplementary material for: Large-Scale Social and Behavior Change Communication Interventions Have Sustained Impacts on Infant and Young Child Feeding Knowledge and Practices: Results of a 2-Year Follow-Up Study in Bangladesh
Source: J Nutr. 2018 Aug 29;148(10):1605–14. doi: 10.1093/jn/nxy147 (PMC6168701; doi:10.1093/jn/nxy147)
Supplement: Supplemental File [file nxy147_supplemental_file.docx]

**Supplementary Table 1. Quality of interpersonal counseling received by mothers with children aged 0-23.9 mo, by program group and survey round^1^**

|  | **Endline 2014 (T2)** | |  | **Follow up 2016 (T3)** | |  |  | |
| --- | --- | --- | --- | --- | --- | --- | --- | --- |
|  | **Intensive** | **Non-intensive** |  | **Intensive** | **Non-intensive** |  | **Intensive** | **Non-intensive** |
| **Indicator** | **(*n* = 1001)** | **(*n* = 1000)** |  | **(*n* = 1200)** | **(*n* = 1200)** |  | **T3 – T2** | **T3 – T2** |
| **Duration of counseling** |  |  |  |  |  |  |  |  |
| Counseling from SS, min | 14.7 + 9.4 | 12.5 + 11.5 |  | 11.0 + 6.2 | 8.7+ 5.8 |  | -3.7^#^ | -3.8 |
| Counseling from SK, min | 14.9 + 9.1 | 13.6 + 11.3 |  | 12.0 + 6.2*** | 9.0 + 5.5 |  | -2.9 | -4.6 |
| Counseling from PK, min | 15.3 + 8.8 | NA |  | 12.0 + 6.3 | NA |  | NA | NA |
| **Content of counseling by SS** |  |  |  |  |  |  |  |  |
| Discussed about nutrition and child feeding in the last visit, % | 74.3*** | 8.2 |  | 68.0*** | 14.6 |  | -6.3 | 6.4 |
| Discussed about BF in the last visit, % | 72.2*** | 7.2 |  | 57.8*** | 13.7 |  | -14.1^#^ | 6.5 |
| Discussed about CF in the last visit, % | 64.1*** | 5.8 |  | 57.3*** | 10.1 |  | -6.8 | 4.3 |
| **Content of counseling by SK** |  |  |  |  |  |  |  |  |
| Discussed about nutrition and child feeding in the last visit, % | 25.4*** | 12.1 |  | 54.8*** | 22.8 |  | 29.4^#^ | 10.7^##^ |
| Discussed about BF in the last visit, % | 24.2*** | 10.9 |  | 52.7*** | 21.7 |  | 28.5^#^ | 10.8^##^ |
| Discussed about CF in the last visit, % | 19.5*** | 8.9 |  | 41.8*** | 15.5 |  | 22.3^#^ | 6.6^#^ |
| **Content of counseling by PK** |  |  |  |  |  |  |  |  |
| Discussed about nutrition and child feeding in the last visit, % | 78.5*** | 0.0 |  | 84.2*** | 0.0 |  | 5.7^#^ | 0.0 |
| Discussed about BF in the last visit, % | 75.9*** | 0.0 |  | 71.9*** | 0.0 |  | -4.0 | 0.0 |
| Discussed about CF in the last visit, % | 69.8*** | 0.0 |  | 74.6*** | 0.0 |  | 4.8 | 0.0 |

^1^ Values are means + SDs or percentages. *^,^**^,^***Different from non-intensive at that time: **P*<0.05, ***P*<0.01, ****P*<0.001. ^#,##^Significant change from endline to follow-up: ^#^*P*<0.05, ^##^*P*<0.01. BF, breastfeeding; CF, complementary feeding; PK, Pushti Kormi (nutrition promoter); SS, Shasthya Sebika (health volunteer); SK, Shasthya Kormi (health worker); T, time.

**Supplementary Table 2. WHO recommended core IYCF indicators, by program group and survey round^1^**

| **Indicator** |  | **Baseline 2010 (T1)** | |  | **Endline 2014 (T2)** | |  | **Follow up 2016 (T3)** | |  | **T3 – T2** | |  | **T3 – T1** | |
| --- | --- | --- | --- | --- | --- | --- | --- | --- | --- | --- | --- | --- | --- | --- | --- |
|  | **Age group, mo** | **Intensive** | **Non-intensive** |  | **Intensive** | **Non-intensive** |  | **Intensive** | **Non-intensive** |  | **Intensive** | **Non-intensive** |  | **Intensive** | **Non-intensive** |
| Early initiation of BF | 0-23 | 63.5 | 64.3 |  | 94.3*** | 75.7 |  | 70.5*** | 54.8 |  | -23.8^##^ | -20.9^###^ |  | 7.0^###^ | -9.6^###^ |
| Exclusive BF | 0-5 | 48.5 | 51.2 |  | 87.6*** | 53.5 |  | 71.3*** | 56.0 |  | -16.3^##^ | 2.5 |  | 22.9^###^ | 4.8 |
| Continued BF at 1 y | 12-15 | 97.5 | 95.5 |  | 96.3 | 93.6 |  | 97.5* | 91.9 |  | 1.2 | -1.7 |  | 0.1 | -3.6 |
| Introduction of solid, semi-solid or soft food | 6-8 | 46.0 | 53.0 |  | 98.7 | 98.1 |  | 85.2* | 74.1 |  | -13.5^#^ | -24.0^##^ |  | 39.3^###^ | 21.1^###^ |
| Minimum diet diversity^2^ | 6-23 | 32.1 | 30.0 |  | 63.6*** | 45.7 |  | 55.3*** | 44.2 |  | -8.3^##^ | -1.5 |  | 23.3^###^ | 14.1^###^ |
| Minimum meal frequency^3^ | 6-23 | 42.1 | 37.2 |  | 75.2*** | 55.3 |  | 84.3*** | 70.4 |  | 9.1^##^ | 15.1^#^ |  | 42.2^###^ | 33.3^###^ |
| Minimum acceptable diet^4^ | 6-23 | 16.0 | 13.6 |  | 50.4*** | 26.2 |  | 49.7*** | 36.5 |  | -0.7 | 10.3 |  | 33.7^###^ | 22.9^###^ |
| Consumption of iron-rich food^5^ | 6-23 | 39.5* | 32.8 |  | 78.4*** | 47.1 |  | 68.0*** | 48.7 |  | -10.4^#^ | 1.6 |  | 28.5^###^ | 15.8^###^ |

^1^ Values are percentages. *^,^**^,^***Different from non-intensive at that time: **P*<0.05, ***P*<0.01, ****P*<0.001. ^#,##,###^Significant change from endline to follow-up or from baseline to follow-up: ^#^*P*<0.05, ^##^*P*<0.01, ^###^*P*<0.001. BF, breastfeeding; IYCF, infant and young child feeding; T, time; WHO, World Health Organization.

^2^ Minimum diet diversity is defined as receiving four or more food groups during the previous day for children 6-23.9 months.

^3^ Minimum meal frequency is defined as 2 times for breastfed infants 6‑8 months; 3 times for breastfed children 9‑23.9 months; and 4 times for non-breastfed children 6‑23.9 months. “Meals” include both meals and snacks and frequency is based on mother’s report.

^4^ Minimum acceptable diet is defined as having at least the minimum dietary diversity and minimum meal frequency during the previous day.

^5^ Iron-rich or iron-fortified foods include flesh foods, commercially fortified foods especially designed for infants and young children that contain iron, or foods fortified in the home with a micronutrient powder containing iron.

**Supplementary Table 3. Breastfeeding knowledge among mothers with children aged 0-23.9 mo, by program group and survey round^1^**

|  | **Baseline 2010 (T1)** | |  | **Endline 2014 (T2)** | |  | **Follow up 2016 (T3)** | |  | **T3 – T2** | |  | **T3 – T1** | |
| --- | --- | --- | --- | --- | --- | --- | --- | --- | --- | --- | --- | --- | --- | --- |
| **Indicator** | **Intensive** | **Non-intensive** |  | **Intensive** | **Non-intensive** |  | **Intensive** | **Non-intensive** |  | **Intensive** | **Non-intensive** |  | **Intensive** | **Non-intensive** |
|  | **(*n* = 1095)** | **(*n* = 1093)** |  | **(*n* = 1001)** | **(*n* = 1000)** |  | **(*n* = 1200)** | **(*n* = 1200)** |  |  |  |  |  |  |
| Baby should be breastfed immediately after birth | 88.0 | 83.3 |  | 95.6 | 93.8 |  | 97.1 | 96.1 |  | 1.5 | 2.3 |  | 9.0^###^ | 12.8^###^ |
| Give baby colostrum | 96.1* | 92.9 |  | 90.9 | 89.3 |  | 98.7** | 97.2 |  | 7.8^##^ | 7.9^#^ |  | 2.6^###^ | 4.3^###^ |
| Baby should be given expressed breastmilk when mother is away | 2.7 | 2.3 |  | 42.2*** | 8.9 |  | 18.6** | 3.6 |  | -23.6^###^ | -5.3 |  | 15.9^###^ | 1.3 |
| Mother knows >1 reason to exclusively breastfeed | 95.8 | 93.5 |  | 99.3* | 98.0 |  | 98.9 | 99.2 |  | -0.4 | 1.2 |  | 3.1^###^ | 5.7^###^ |
| Baby <6 mo should not be given water in hot weather | 27.2** | 36.6 |  | 76.1*** | 36.4 |  | 63.6*** | 47.7 |  | -12.5^#^ | 11.3 |  | 36.4^###^ | 11.1^###^ |
| Continue BF until child is 24 mo old | 92.0 | 92.7 |  | 91.6* | 85.2 |  | 93.5 | 91.1 |  | 1.9 | 5.9 |  | 1.5 | -1.6 |
| Continue BF even if the mother is pregnant | 64.5 | 73.9 |  | 89.0*** | 73.0 |  | 80.2 | 84.0 |  | -8.8 | 11.0^##^ |  | 15.7^###^ | 10.1^###^ |
| Continue BF even if the mother is ill | 79.1 | 83.3 |  | 94.2*** | 85.1 |  | 81.8* | 91.9 |  | -12.4^#^ | 6.8^#^ |  | 2.7 | 8.7^###^ |
| **BF knowledge score (range: 0-8)** | 5.5 + 1.1 | 5.6 + 1.2 |  | 6.8 + 1.1*** | 5.7 + 1.1 |  | 6.3 + 1.1^+^ | 6.1 + 0.9 |  | -0.5^##^ | 0.4^##^ |  | 0.9^###^ | 0.5^###^ |

^1^ Values are means + SDs or percentages. ^+,^*^,^**^,^***Different from non-intensive at that time: ^+^*P*<0.1, **P*<0.05, ***P*<0.01, ****P*<0.001. ^#,##,###^Significant change from endline to follow-up or from baseline to follow-up: ^#^*P*<0.05, ^##^*P*<0.01, ^###^*P*<0.001. BF, breastfeeding; T, time.

**Supplementary Table 4. Complementary feeding knowledge among mothers with children aged 0-23.9 mo, by program group and survey round^1^**

|  | **Baseline 2010 (T1)** | |  | **Endline 2014 (T2)** | |  | **Follow up 2016 (T3)** | |  | **T3 – T2** | |  | **T3 – T1** | |
| --- | --- | --- | --- | --- | --- | --- | --- | --- | --- | --- | --- | --- | --- | --- |
| **Indicator** | **Intensive** | **Non-intensive** |  | **Intensive** | **Non-intensive** |  | **Intensive** | **Non-intensive** |  | **Intensive** | **Non-intensive** |  | **Intensive** | **Non-intensive** |
|  | **(*n* = 1095)** | **(*n* = 1093)** |  | **(*n* = 1001)** | **(*n* = 1000)** |  | **(*n* = 1200)** | **(*n* = 1200)** |  |  |  |  |  |  |
| Knows a child should start receiving liquids at 6 mo | 60.6** | 73.8 |  | 93.5*** | 81.7 |  | 83.9* | 75.5 |  | -9.6^#^ | -6.2 |  | 23.3^###^ | 1.7 |
| Knows a child should start receiving food at 6 mo | 78.0 | 72.7 |  | 87.0** | 76.2 |  | 96.3 | 96.3 |  | 9.3^##^ | 20.1^##^ |  | 18.3^###^ | 23.6^###^ |
| Meal frequency at 7-8.9 mo | 92.0 | 82.9 |  | 93.3 | 90.8 |  | 98.5 | 96.7 |  | 5.2^##^ | 5.9 |  | 6.5^###^ | 13.8^###^ |
| Meal frequency at 9-11.9 mo | 59.3 | 50.6 |  | 86.7 | 88.2 |  | 95.0 | 92.2 |  | 8.3^##^ | 4.0 |  | 35.7^###^ | 41.6^###^ |
| Meal frequency at 12-23.9 mo | 7.3 | 17.0 |  | 97.6 | 96.5 |  | 99.1 | 98.9 |  | 1.5^#^ | 2.4 |  | 91.8^###^ | 81.9^###^ |
| Feeding during illness | 98.0** | 99.2 |  | 97.2 | 97.3 |  | 99.5 | 99.7 |  | 2.3^##^ | 2.4^###^ |  | 1.5^##^ | 0.5 |
| Feeding after illness | 79.1 | 79.0 |  | 81.5** | 72.6 |  | 83.3 | 86.6 |  | 1.8 | 14.0^#^ |  | 4.2^#^ | 7.6^###^ |
| Knows ≥1 way to encourage CF | 93.5 | 92.4 |  | 97.5 | 96.4 |  | 74.8 | 72.3 |  | -22.7^###^ | -24.1^##^ |  | -18.7^###^ | -20.2^###^ |
| **CF knowledge score (range: 0-8)** | 5.7 + 1.0 | 5.7 + 1.0 |  | 7.3 + 0.9** | 7.0 + 1.1 |  | 7.3 + 0.8^+^ | 7.2 + 0.9 |  | 0.0 | 0.2^###^ |  | 1.6^###^ | 1.5^###^ |

^1^ Values are means + SDs or percentages. ^+,^*^,^**^,^***Different from non-intensive at that time: ^+^*P*<0.1, **P*<0.05, ***P*<0.01, ****P*<0.001. ^#,##,###^Significant change from endline to follow-up or from baseline to follow-up: ^#^*P*<0.05, ^##^*P*<0.01, ^###^*P*<0.001. CF, complementary feeding; T, time.
